# Human papillomavirus type 16 E6 suppresses microRNA-23b expression in human cervical cancer cells through DNA methylation of the host gene C9orf3

**DOI:** 10.18632/oncotarget.14555

**Published:** 2017-01-06

**Authors:** Chi Lam Au Yeung, Tsun Yee Tsang, Pak Lun Yau, Tim Tak Kwok

**Affiliations:** ^1^ School of Biomedical Sciences, Faculty of Medicine, The Chinese University of Hong Kong, Hong Kong SAR, China; ^2^ Department of Gynecologic Oncology and Reproductive Medicine, The University of Texas MD Anderson Cancer Center, Houston, TX, USA

**Keywords:** HPV-16 E6, miRNA-23b, DNA methylation, C9orf3, cervical cancer

## Abstract

Oncogenic protein E6 of human papillomavirus type 16 (HPV-16) is believed to involve in the aberrant methylation in cervical cancer as it upregulates DNA methyltransferase 1 (DNMT1) through tumor suppressor p53. In addition, DNA demethylating agent induces the expression of one of the HPV-16 E6 regulated microRNAs (miRs), miR-23b, in human cervical carcinoma SiHa cells. Thus, the importance of DNA methylation and miR-23b in HPV-16 E6 associated cervical cancer development is investigated. In the present study, however, it is found that miR-23b is not embedded in any typical CpG island. Nevertheless, a functional CpG island is predicted in the promoter region of C9orf3, the host gene of miR-23b, and is validated by methylation-specific PCR and bisulfite genomic sequencing analyses. Besides, c-MET is confirmed to be a target gene of miR-23b. Silencing of HPV-16 E6 is found to increase the expression of miR-23b, decrease the expression of c-MET and thus induce the apoptosis of SiHa cells through the c-MET downstream signaling pathway. Taken together, the tumor suppressive miR-23b is epigenetically inactivated through its host gene C9orf3 and this is probably a critical pathway during HPV-16 E6 associated cervical cancer development.

## INTRODUCTION

MicroRNA (miR), a class of small non-coding single-stranded RNA of 19 to 24 nucleotides in length, is recently believed to participate in the development of cancer, including cervical cancer. The main function of miR is to repress the expression of target mRNA by either cleavage or translational silencing. The silencing action depends on the degree of complementation of miRNA sequence with the 3’-UTR of target mRNAs [1–3].

“Knowing” the mechanisms that regulate the expression of miRNAs is critical in understanding the role of miRs in cervical cancer development. So far, the molecular mechanisms however remain largely unknown. Several microarray profiling studies showed that the expression pattern of a large number of miRNAs can be attributed to the regulatory sequences present in their promoters [4]. Therefore, miRNAs may probably be controlled by nuclear transcription factors through either transactivation or transrepression in ways similar as that for the protein-coding genes [5]. The transcription factors, such as p53, c-myc, E2F and NFκB, were reported to be involved in the regulation of miRNA expressions [5–8]. On the other hand, the regulation of miRNAs may also be at the post-transcriptional level, such as miRNA maturation process. Discrepancies among the levels of primary transcript, precursor and mature miRNA species have been reported. The biogenesis of mature miRNAs mainly involves two RNase III enzymes, Drosha and Dicer. Previous studies showed that a large fraction of miRNA might be regulated at the Drosha processing step [4, 9].

Epigenetic modification such as DNA hypermethylation often results in the silencing of genes while aberrant DNA methylation is detected in many cancers. Thus, epigenetic silencing may also be one of the possible mechanisms that contribute to miRNA regulation, especially for miRNAs that with tumor suppressive function, such as miR-124a and 127 [10, 11]. Oncogenic viruses, such as human papillomavirus (HPV), are often thought to be involved in the alteration of DNA methylation in the host cells, a process appears to be critical in cancer progression [12–14]. In fact, DNA methyltransferase 1 (DNMT1), a major enzyme for DNA methylation, was found to be overexpressed in human cervical cancer [15].

Nearly half of the miRNAs are located within the introns of protein-coding genes or non-coding transcriptional units, which are known as the host genes. miR-23b is located in the intron 14 of the host gene C9orf3 on chromosome 9. Recent studies showed that the expression of miR-23b could be regulated via the upstream promoter region of the host gene [5, 16] and may also be epigenetically regulated.

One of the target genes identified for miR-23b is c-MET, which is believed to act as a protooncogene and participate in cell proliferation [17, 18]. c-MET was also overexpressed in many human solid tumors including uterine cervix carcinomas and its overexpression served as an important prognostic indicator [19, 20].

In the present study, HPV-16 E6 was confirmed to regulate miR-23b indirectly through the DNA methylation of host gene C9orf3 and thus induce c-MET and inhibit apoptosis in cervical cancer cells. The uniqueness and the significance for indirect epigenetic regulation pathway of miRs, from the host gene C9orf3 to miR-23b, is worth further investigation.

## RESULTS

### Reduced miR-23b expression in DNMT knockout cells

**Figure 1:**
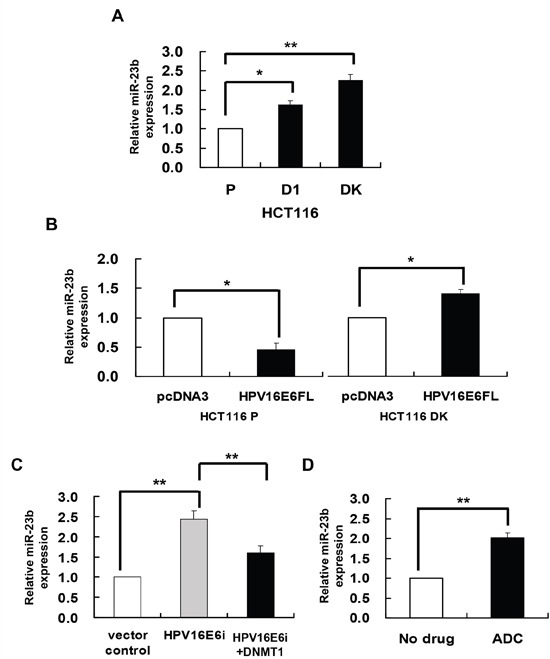
DNMT1 mediated the HPV-16 E6 suppression of miR-23 miR-23b expression level was determined by quantitative RT-PCR analysis. **A**. the miR-23b expression level in HCT116 parental, D1 and DK cells. **B**. HCT116 parental or DK cells were transiently transfected with pcDNA3/HPV16E6cDNA (HPV16E6FL) or pcDNA3 empty vector for 24 h. **C**. SiHa cells were transiently transfected with pSilencer/HPVE6siRNA (HPV16E6i) alone or co-transfected with pGFP-C3/DNMT1cDNA (HPV16E6i+DNMT1) for 24 h. The cells were transfected with pSilencer empty vector in parallel in which miR-23b expression level was designated as 1.0. **D**. SiHa cells were treated with 5 μM 5-aza-2’-deoxycytidine (ADC) for 24 h. Results were average from at least three separate experiments. Mean ± SD. *p<0.05, **p<0.01.

miR-23b is suggested to be epigenetically regulated in the previous study. miR-23b together with a number of miRs were found to be overexpressed in human colorectal carcinoma HCT116 double DNMT1 and DNMT3b knockout (DK) cells as compared to the HCT116 parental cells [21]. It is therefore believed that these miRs may be regulated epigenetically. miR-23b was confirmed to be overexpressed in DK cells by quantitative RT-PCR analysis in the present study (Figure 1A). In addition, similar to DK cells, the miR was also overexpressed in DNMT1 knockout (D1) cells (Figure 1A), suggesting that DNMT1 may suppress the expression of miR-23b, presumably by methylation, in cells.

### DNMT1 mediated HPV-16 E6 suppression of miR-23b

HPV-16 E6 was previously found to upregulate DNMT1 in human cervical cancer cells [22]. Therefore, it is hypothesized that HPV-16 E6 may act through methylation to regulate the expression of miR-23b. To investigate the regulatory role of HPV-16 E6/DNMT1 in miR-23b expression, ectopic expression of HPV-16 E6 in HCT116 parental and DK cells was carried out by transfecting the cells with HPV-16 E6 cDNA [22]. HPV-16 E6 suppressed miR-23b expression in HCT116 parental cells but failed in the DK cells, indicating that the suppression effect of HPV-16 E6 on miR-23b was mediated through DNMTs (Figure 1B). On the other hand, in HPV-16 E6 positive human cervical cancer SiHa cells, knockdown of HPV-16 E6 by E6 siRNA increased the miR-23b level in cells. Such increase, however, was lessened by 84% upon the co-transfection with DNMT1 cDNA (Figure 1C). This further indicated that DNMT1 might mediate the suppressive effect of HPV-16 E6 on miR-23b.

### Lack of CG rich region in the miR-23b upstream sequence

DNMT1 is known to regulate the expression of genes through DNA methylation. Whether DNMT1 may mediate the HPV-16 E6 effect on miR-23b through DNA methylation is to be examined. By quantitative RT-PCR analysis, a 2-fold increase in the expression of miR-23b was observed in SiHa cells treated with DNA demethylating agent, 5-aza-2’deoxycytidine (ADC), indicating the likely involvement of DNA methylation in the regulation of miR-23b (Figure 1D).

By then, the upstream sequence of miR-23b spanning from 1kb upstream to the mature miR-23b was analyzed for the presence of any CG rich region, where presumably DNA methylation may occur. Using the conventional online algorithms for CpG island prediction, such as Methprimer, no typical CpG island (200 nucleotides or more with CG content greater than 50%) was detected. Despite the failure in the prediction of putative CpG island in the miR-23b upstream sequence, the possibility of the sequence being methylated was still examined by the methylation-specific PCR (MSP) analysis. No significant difference was however detected between the ADC-treated cells and the untreated cells or the HPV-16 E6 knockdown cells and the pSilencer transfected control cells (Supplementary Figure 1). Also, when SiHa cells were transfected with the luciferase reporter containing the miR-23b upstream sequence, the reporter activities in cells with or without the ADC treatment were found to be similar. All the results suggested the lack of CG rich region in the upstream sequence of miR-23b and the unlikely involvement of this sequence in the DNA methylation regulation of miR-23b.

### HPV-16 E6 and DNA methylation decreased C9orf3 mRNA expression

**Figure 2:**
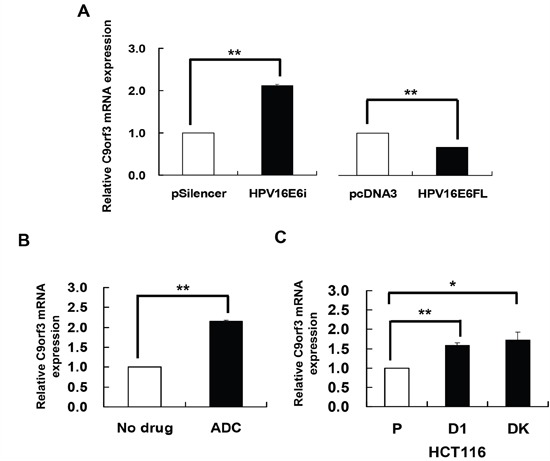
HPV-16 E6 silencing and DNA demethylation increased the expression of C9orf3 mRNA C9orf3 mRNA expression level was determined by quantitative RT-PCR analysis. **A**. SiHa cells were transiently transfected with pSilencer/HPV16E6siRNA (HPV16E6i) or pcDNA3/HPV16E6cDNA (HPV16E6FL) for 24 h. The cells were transfected with pSilencer or pcDNA3 empty vector in parallel. **B**. SiHa cells were treated with 5 μM 5-aza-2’-deoxycytidine (ADC) for 24 h. **C**. C9orf3 mRNA expression level in HCT116 parental, D1 and DK cells. Results were average from at least three separate experiments. Mean ± SD. *p<0.05, **p<0.01.

miR-23b is an intronic miRNA and the host gene is C9orf3. Although intronic miRNAs can be regulated by the miRNAs’ own promoters, they may also be co-regulated with their host genes [23–25]. Thus, HPV-16 E6 may regulate miR-23b, an intronic miRNA, indirectly through the methylation of its host gene rather than directly the miRNA's upstream sequence. To test this hypothesis, the effects of HPV-16 E6 and ADC on the expression of the host gene C9orf3 were first determined. Knockdown of HPV-16 E6 by HPV-16 E6 siRNA resulted in an increase while full-length E6 cDNA transfection led to a decrease in the expression of C9orf3 mRNA in SiHa cells (Figure 2A).

On the other hand, ADC treatment increased the expression of C9orf3 mRNA (Figure 2B) as well as the activity of the reporter containing the C9orf3 upstream sequence in SiHa cells. Furthermore, in HCT116 DK and D1 cells, the level of C9orf3 mRNA was elevated as compared with the parental cells (Figure 2C). The results therefore suggested that HPV-16 E6 may probably epigenetically regulate C9orf3 and thus miR-23b by DNMTs.

### HPV-16 E6 induced methylation of C9orf3 promoter through DNMT1

**Figure 3:**
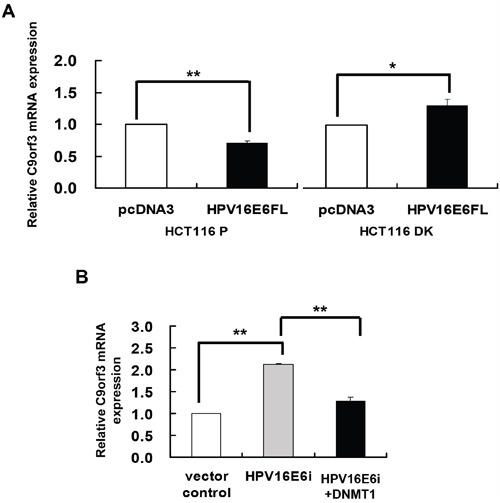
DNMT1 mediated the HPV-16 E6 suppression of C9orf3 mRNA expression C9orf3 mRNA expression level was determined by quantitative RT-PCR analysis. **A**. HCT116 parental or DK cells were transiently transfected with pcDNA3/HPV16E6cDNA (HPV16E6FL) or pcDNA3 empty vector for 24 h. **B**. SiHa cells were transiently transfected with pSilencer/HPVE6siRNA (HPV16E6i) alone or co-transfected with pGFP-C3/DNMT1cDNA (HPV16E6i+DNMT1) for 24 h. The cells were transfected with pSilencer empty vector in parallel in which C9orf3 mRNA expression level was designated as 1.0. Results were average from at least three separate experiments. Mean ± SD. *p<0.05, **p<0.01.

To elucidate the role of HPV-16 E6 in DNA methylation regulation of C9orf3, HPV-16 E6 was overexpressed in HCT116 parental and DK cells. Similar to that observed for miR-23b (Figure 1B), HPV-16 E6 suppressed C9orf3 mRNA in the parental cells but not in the DK cells (Figure 3A). On the other hand, transfection with HPV-16 E6 siRNA increased the level of C9orf3 mRNA while co-transfection with DNMT1 cDNA offset such increase in SiHa cells (Figure 3B). These indicated that HPV-16 E6 controlled the expression of C9orf3 in the same manner as that of miR-23b.

**Figure 4:**
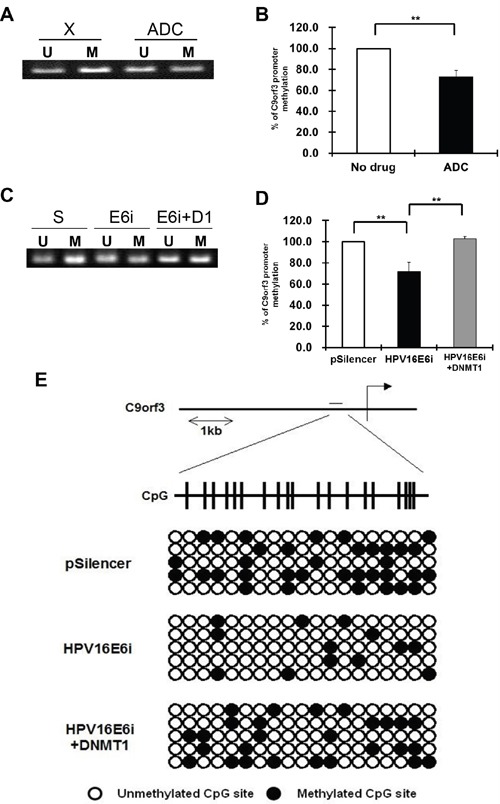
The methylation status of the C9orf3 promoter region in SiHa cells **A** and **B**. the cells were either untreated (X) or treated with 5 μM 5-aza-2’-deoxycytidine (ADC) for 24 h. A, the PCR products amplified by unmethylation-specific primers (U) and methylation-specific primers (M) were revolved in 2% agarose gel. A representative experiment was shown while similar results were obtained from at least three separate experiments. B, the percentage of C9orf3 promoter methylation was shown. Mean ± SD. **p<0.01. **C** and **D**. the cells were transiently transfected with pSilencer/HPV16E6siRNA (E6i) alone or co-transfected with pGFP-C3/DNMT1cDNA (E6i+D1) for 24 h. The cells were transfected with pSilencer empty vector (S) in parallel. (C) the PCR products amplified by unmethylation-specific primers (U) and methylation-specific primers (M) were revolved in 2% agarose gel. A representative experiment was shown while similar results were obtained from at least three separate experiments. (D) the percentage of C9orf3 promoter methylation was shown. Mean ± SD. **p<0.01. **E**. genomic bisulfite sequencing of C9orf3 promoter region was performed. The cells were transiently transfected with pSilencer/HPV16E6siRNA (HPV16E6i) alone or co-transfected with pGFP-C3/DNMT1cDNA (HPV16E6i+DNMT1) for 24 h. The cells were transfected with pSilencer empty vector in parallel. The position of the CpG island in C9orf3 promoter region was indicated. Open and closed circles represent unmethylated and methylated CpG sites, respectively.

Using online CpG island prediction algorithms, a putative CpG island was identified in the region 1 kb upstream from the transcription start site of C9orf3 mRNA. MSP and bisulfite sequencing analyses were therefore performed to validate the functional roles of HPV-16 E6 and DNMT1 in the methylation of this C9orf3 upstream sequence. By MSP analysis, hypomethylation in the C9orf3 upstream sequence was found in SiHa cells after the treatment with ADC as well as HPV-16 E6 knockdown (Figure 4A to 4D). Furthermore, the level of hypomethylation after HPV-16 E6 knokdown was reduced with the over-expression of DNMT1. (Figure 4C and 4D) The MSP results were confirmed by bisulfite sequencing analysis. The methylation of selected CpG islands was less in SiHa cells after HPV-16 E6 knockdown but was partly restored by DNMT1 overexpression (Figure 4E). This verified that HPV-16 E6 induced the methylation of the C9orf3 promoter through DNMT1.

### Induction of C9orf3 promoter methylation suppressed miR-23b expression

**Figure 5:**
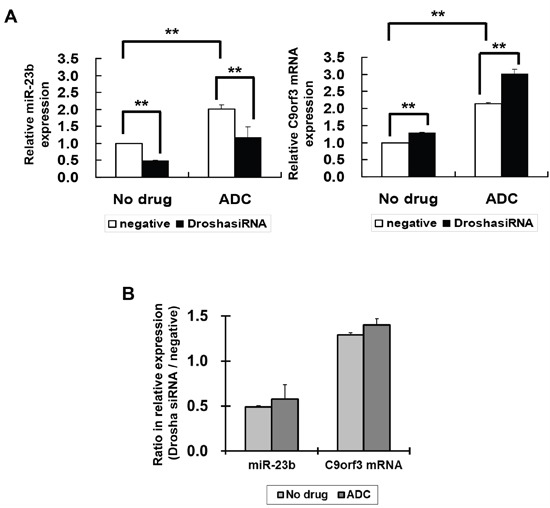
The effect of Drosha and 5-aza-2’-deoxycytidine on miR-23b and C9orf3 mRNA expressions in SiHa cells **A**. The cells were transiently transfected with Drosha siRNA or negative siRNA for 24 h followed by 24-hour treatment of 5-aza-2’-deoxycytidine (ADC). miR-23b and C9orf3 mRNA expression levels were then determined by quantitative RT-PCR analysis. Results were average from at least three separate experiments. Mean ± SD. **p<0.01. **B**. the ratio (Drosha siRNA / negative) for the relative expression of miR-23b or C9orf3 mRNA in cells with or without the ADC treatment.

The level of mature miRNA may be affected by the processing of miRNA from the host gene. Therefore, to confirm whether the effect of HPV-16 E6 on C9orf3 promoter methylation will lead to its effect on miR-23b expression, the role of miRNA processing should also be examined. Drosha is a RNase III that plays an initiative role in miRNA processing. Silencing of Drosha by Drosha-specific siRNA led to a nearly 50% reduction in the expression of miR-23b in SiHa cells, whereas C9orf3 mRNA expression was slightly increased (Figure 5A). This indicated the role of Drosha in the processing of host gene C9orf3 into miR-23b. The ADC treatment upregulated both miR-23b and C9orf3 mRNA in SiHa cells and both effects appeared to be unchanged even with Drosha silencing (Figure 5A). The ratio between the relative expression of miR-23b or C9orf3 mRNA in Drosha and control siRNA transfected cells was not affected by the ADC treatment (Figure 5A and 5B). Taken together, the results supported the idea that HPV-16 E6 may suppress the expression of miR-23b through the methylation of C9orf3 promoter.

### c-MET as a gene target of miR-23b

**Figure 6:**
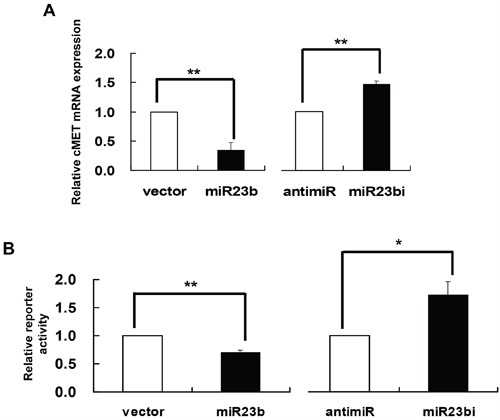
c-MET as a gene target of miR-23b **A**. SiHa cells were transiently transfected with miR-23b precursor vector (miR23b) or anti-miR-23b inhibitor (miR23bi), respectively, for 24 h. The cells were transfected with empty vector or anti-miR-negative control in parallel. c-MET mRNA expression level was determined by quantitative RT-PCR analysis. **B**. SiHa cells were transiently transfected with pMIR-REPORT/cMET3’UTR together with miR-23b precursor vector (miR23b) or anti-miR-23b inhibitor (miR23bi) for 24 h followed by dual-luciferase reporter assay. The cells were co-transfected with empty vector or anti-miR-negative control in parallel. Results were average from at least three separate experiments. Mean ± SD. *p<0.05, **p<0.01.

c-MET, also known as hepatocyte growth factor receptor (HGFR), is a protooncogene reported to be over-expressed in uterine cervical cancer [19]. Notably, it was found to be a gene target of miR-23b [26]. To verify this relationship in human cervical carcinoma cells, c-MET mRNA and protein levels were assessed in SiHa cells with miR-23b precursor vector or anti-miR-23b inhibitor transfection [27]. By quantitative RT-PCR analysis, the c-MET mRNA was decreased to 34.8% or increased to 146.4% of the control in SiHa cells transfected with miR-23b precursor vector or anti-miR-23b inhibitor respectively (Figure 6A). The interaction between miR-23b and c-MET mRNA was further investigated by dual-luciferase reporter assay. SiHa cells were transfected with the reporter construct containing the 3’-UTR of c-MET mRNA [26] together with either miR-23b precursor vector or anti-miR-23b inhibitor. The precursor vector suppressed the reporter activity by 29.7% and the inhibitor raised that by 71.5% (Figure 6B). Therefore, c-MET is confirmed to be the gene target of miR-23b in SiHa cells.

### HPV-16 E6 downregulated c-MET through DNMT1 and miR-23b

**Figure 7:**
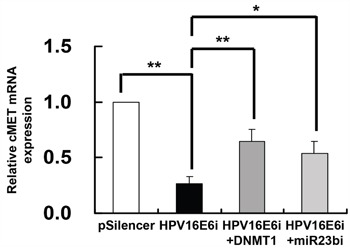
DNMT1 and miR-23b mediated the effect of HPV-16 E6 knockdown on c-MET mRNA expression in SiHa cells The cells were transiently transfected with pSilencer/HPV16E6siRNA (HPV16E6i) alone or co-transfected with either pGFP-C3/DNMT1cDNA (HPV16E6i+DNMT1) or anti-miR-23b inhibitor (HPV16E6i+miR23bi) for 24 h. The cells were transfected with pSilencer empty vector in parallel. c-MET mRNA expression level was determined by quantitative RT-PCR analysis. Results were average from at least three separate experiments. Mean ± SD. *p<0.05, **p<0.01.

Since HPV-16 E6 downregulated miR-23b through methylation of its host gene C9orf3 and c-MET was identified as a target of miR-23b, it was hypothesized that HPV-16 E6 may repress the expression of c-MET through DNMT1 and miR-23b. To prove that, SiHa cells were transfected with HPV-16 E6 siRNA alone or co-transfected together with either DNMT1 cDNA or anti-miR-23b inhibitor. The expression of c-MET mRNA was reduced to 26.5% of the control with only HPV-16 E6 siRNA transfection but 64.4% or 54.0% when co-transfected with DNMT1 cDNA or anti-miR-23b inhibitor respectively (Figure 7). This supported a link from HPV-16 E6, DNMT1, miR-23b to c-MET.

### HPV-16 E6 silencing promoted apoptosis in SiHa cells through miR-23b and c-MET

**Figure 8:**
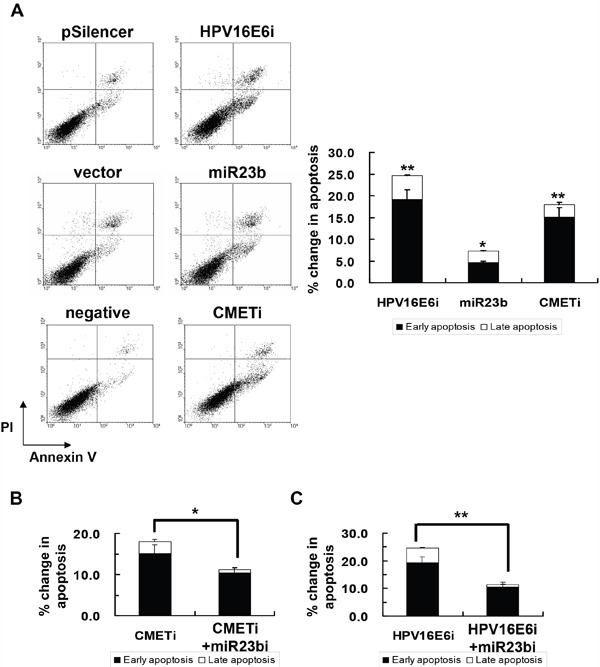
The effect of HPV-16 E6, miR-23b and c-MET on apoptotic cell death of SiHa cells **A**. the cells were transiently transfected with pSilencer/HPV16E6siRNA (HPV16E6i), miR-23b precursor vector (miR23b) or c-MET siRNA (CMETi). The cells were transfected with empty pSilencer vector (pSilencer), empty vector (vector) or negative siRNA (negative), respectively, in parallel. Annexin V binding assay was performed 48 h after transfection to determine apoptotic cell death. A representative experiment was shown while similar results were obtained from at least three separate experiments. The percentage changes in early and late apoptosis were calculated by subtracting the percentage of apoptotic cells in the group transfected with empty pSilencer vector, empty vector or negative siRNA from that in the corresponding group transfected with pSilencer/HPV16E6siRNA, miR23b precursor vector or c-MET siRNA, respectively, and were shown as the bar chart in the right panel. **B**. the cells were transiently transfected with anti-miR-23b inhibitor together with c-MET siRNA (miR23bi+CMETi) or **C**. pSilencer/HPV16E6siRNA together with anti-miR-23b inhibitor (HPV16E6i+miR23bi) for 48 h followed by the annexin V binding assay to determine apoptotic cell death. The percentages of early and late apoptotic cells were shown in the bar chart. Results were average from at least three separate experiments. Mean ± SD. *p<0.05, **p<0.01.

Knockdown of HPV-16 E6 was reported to induce apoptosis in human cervical carcinoma cells [28, 29] and the effect was observed in SiHa cells (Figure 8A). To see if HPV-16 E6 may act through miR-23b and c-MET in regulation of apoptosis, the roles of c-MET and miR-23b in apoptotic induction during cervical cancer development were investigated. Knockdown of c-MET by c-MET siRNA resulted in an 18% increase in apoptosis in SiHa cells. Similarly, overexpression of miR-23b led to an 8% increase in apoptosis in SiHa cells. (Figure 8A)

In addition, upon co-transfection together with anti-miR-23b inhibitor, the apoptosis in SiHa cells with HPV-16 E6 siRNA transfection was decreased from 25% to 11% (Figure 8B). In the case of c-MET siRNA transfection, the apoptosis was decreased from 18% to 11% (Figure 8C). This suggested the counteracting action between miR-23b and c-MET in apoptotic induction. This demonstrated that miR-23b and c-MET might mediate the effect of HPV-16 E6 on apoptotic regulation in SiHa cells.

**Figure 9:**
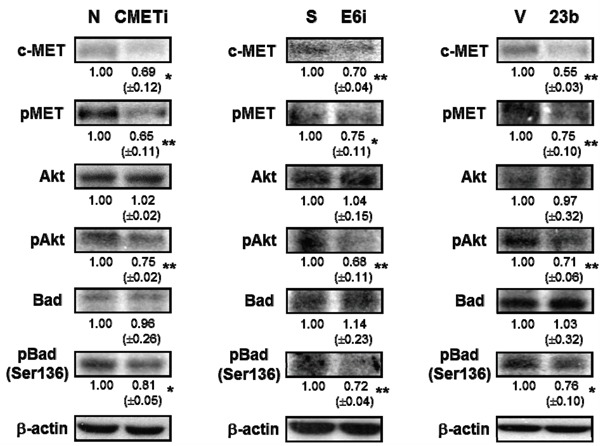
The effect of HPV-16 E6, miR-23b and c-MET on the expressions of proteins in the c-MET/Akt pathway in SiHa cells The cells were transiently transfected with c-MET siRNA (cMETi), pSilencer/HPV16E6siRNA (E6i) or miR-23b precursor vector (23b) for 24 h. The cells were transfected with empty negative siRNA (N), pSilencer vector (S) or empty vector (V), respectively, in parallel. c-MET, phospho-MET (pMET), Akt, phospho-Akt (pAkt), Bad and phospho-Bad (Ser-136) (pBad (Ser-136)) protein expression levels were determined by western blot analysis. β-actin was served as the loading control. The relative expression levels were first normalized with that of β-actin and shown beneath the bands. Mean ± SD. *p<0.05, **p<0.01. A representative experiment was shown while similar results were obtained from at least three separate experiments.

As the receptor of hepatocyte growth factor (HGF), c-MET was believed to regulate apoptosis through the Akt signaling pathway [18]. Phosphorylation of c-MET and Akt subsequently inhibits the activity of proapoptotic Bad by Serine-136 phosphorylation [30, 31]. Transfection with c-MET siRNA resulted in the dephosphorylation of c-MET and Akt in SiHa cells. This in turn deactivated the proapoptotic Bad through dephosphorylation and may lead to apoptotic induction. The c-MET/Akt deactivation cascade (Figure 9) was also demonstrated in SiHa cells transfected with miR-23b precursor vector or HPV-16 E6 siRNA, indicating HPV-16 E6 silencing promoted apoptosis through the miR-23b-mediated c-MET/Akt deactivation pathway.

## DISCUSSION

### HPV-16 E6 regulated miR-23b through DNMT1

Cancer cells generally exhibit a lower miRNA expression level compared to normal cells [21]. These miRNAs probably demonstrated a tumor suppressive function and could be regulated by DNA methylation. Oncogenic protein E6 of HPV-16 upregulated DNMT1 through the repression of tumor suppressor p53 [22]. This indicated that HPV-16 E6 may likely be responsible for the aberrant methylation in cervical cancer. Besides, HPV-16 E6 was reported to be involved in the miRNA deregulation during cervical cancer development [32, 33]. Amongst, miR-23b was one of the miRNAs suppressed by HPV-16 E6 in SiHa cells [27]. Thus, it was hypothesized that miR-23b might be regulated by HPV-16 E6 through DNMT1. This was confirmed as: (i) the miR-23b expression was decreased in SiHa cells treated with DNA methyltransferases inhibitor, 5-aza-2’-deoxycytidine (ADC) (Figure 1D); (ii) HPV-16 E6 lacked the ability to suppress miR-23b expression in HCT116 DK cells (Figure 1B); and (iii) DNMT1 overexpression in SiHa cells counteracted the effect of HPV-16 E6 knockdown on miR-23b expression (Figure 1C).

### HPV-16 E6 downregulated miR-23b through hypermethylation of C9orf3 promoter

Some of the epigenetically regulated intronic miRNAs are embedded in CpG islands, such as miR-1-1 [34], miR-124a [11] and miR-199a [35]. As shown in the present study, miR-23b, however, is not embedded in any typical CpG island. On the other hand, a CG rich region was previously predicted in the promoter of its host gene C9orf3 [36]. Also, a putative CpG island located within 1 kb upstream to the transcription start site of C9orf3 mRNA was predicted by online prediction algorithm MethPrimer and later on was verified by both methylation-specific PCR (MSP) and bisulfite sequencing analyses. Thus, HPV-16 E6 was believed to regulate miR-23b through the methylation of its host gene C9orf3 as intronic miRNAs may be transcribed as part of the host genes [5, 37, 38]. It is supported as miR-23b responded to epigenetic silencing in the same way as C9orf3 mRNA, for examples, the effect of HPV-16 E6 in HCT116 DK cells (Figure 1B and 3A) and the effect of ADC treatment (Figure 1D and 2B), even though typical CpG island was not detected in the upstream sequence of miR-23b. Most importantly, HPV-16 E6 knockdown upregulated miR-23b as well as C9orf3 mRNA in SiHa cells and both worked antagonistically with DNMT1 (Figure 1C and 3B).

### HPV-16 E6 silencing induced apoptosis through miR-23b and c-MET downstream signaling pathway

The protooncogene c-MET is a potential therapeutic target for cancers [39]. It is often overexpressed and mutated in various human solid tumors [17, 40, 41], including cervical carcinomas [19], while its overexpression was thought to be associated with the infection of oncogenic HPVs [42]. Recent studies showed that c-MET was the gene target of different methylation-regulated miRNAs, for examples, miR-1-1 [34] and miR-199a* [43]. Similar to previously reported, c-MET was identified as a target of miR-23b in the present study, which is also methylation-regulated. The c-MET mRNA level was suppressed by miR-23b presumably by direct interaction between the miR and the 3’UTR of c-MET mRNA (Figure 6). In addition, the counteraction between HPV-16 E6 siRNA and miR-23b inhibitor on c-MET mRNA expression further verified the regulatory pathway from HPV-16 E6 to miR-23b/c-MET (Figure 7).

As a result, HPV-16 E6 may act through miR-23b and then c-MET to regulate apoptosis in human cervical cancer cells as: (i) silencing of c-MET was shown to induce apoptosis in SiHa cells (Figure 8A); (ii) miR-23b, the negative regulator of c-MET, also triggered apoptotic induction (Figure 8A); (iii) the anti-miR-23b inhibitor reduced the apoptosis induced by knockdown of either c-MET or HPV-16 E6 (Figure 8B). c-MET was shown to activate diverse intracellular signaling pathways [44]. One of the major effectors downstream of c-MET is Akt [43]. Akt is responsible for the promotion of survival through the phosphorylation of Bad, thereby preventing cell death [45]. Reduced levels of phospho-Akt and phospho-Bad were demonstrated in SiHa cells transfected with HPV-16 E6 siRNA, miR-23b precursor vector or c-MET siRNA, indicating the increased susceptibility of cells to apoptotic cell death (Figure 9). In addition, transfection with HPV-16 E6 siRNA, miR-23b precursor vector or c-MET siRNA also reduces cell proliferation of SiHa cells (Supplementary Figure 2), which is also known to be regulated by c-MET.

### p53 regulated miR-23b both transcriptionally and epigenetically

Epigenetic regulation of miR-23b by DNMT1 through its host gene C9orf3 was shown in the present study. Also, the critical role of HPV-16 E6 in the transcriptional regulation of DNMT1 and miR-23b through direct interaction of tumor suppressor p53 acting on their promoters were previously reported [22, 27]. The importance of these two regulatory pathways on miR-23b expression in human cervical cancer cells was therefore examined. The expressions of miR-23b and C9orf3 mRNA in the ADC-treated p53 knockdown SiHa cells were found to be higher than those in the ADC-untreated p53 knockdown cells (Supplementary Figure 3). This indicated the presence of the epigenetic regulation of miR-23b through its host gene in the cervical cancer development. However, further investigation is required for determining the dominance of each pathway.

To sum up, this study introduces another possible unique regulatory mechanism for miR-23b, which appears to be another major pathway during cervical carcinogenesis. Knockdown of oncogenic HPV-16 E6 protein reduces DNMT1 and thus increases miR-23b expression through hypomethylation of the host gene C9orf3. This in turn decreases the expression of c-MET and deactivates the subsequent signaling pathway to induce apoptosis in human cervical cancer cells. The results suggest that targeting miR-23b may be effective in curing cervical cancer.

## MATERIALS AND METHODS

### Cell lines and reagents

Human cervical carcinoma SiHa cells, human colorectal carcinoma HCT116 parental cells (P), DNMT1 gene knockout HCT116 cells (D1) and double DNMT1 and DNMT3b genes knockout HCT116 cells (DK) were cultured in Dulbecco's modified Eagle's medium (DMEM) supplemented with 10% fetal bovine serum and 2 mmol/L L-glutamine (Invitrogen, CA, USA). All cells were incubated in humidified incubator with 10% CO_2_ at 37°C.

### Construction of HPV-16 E6 siRNA and full-length HPV-16 E6 cDNA expression vectors

The construction of HPV-16 E6 siRNA and full-length HPV-16 E6 cDNA expression vectors were performed as previously described [27]. All plasmids were transfected into cells using Lipofectamine 2000 (Invitrogen) for 24 h.

### Treatment with DNA demethylating agent

Cells were treated with 5 μM DNA demethylating agent, 5-aza-2’deoxycytidine (ADC; Calbiochem) for 24 h either before sample extraction or 24 h after transfection.

### Quantitative real-time RT-PCR analysis

Total RNA was extracted using TRIreagent (Molecular Research Centre, OH, USA). For mRNA expression analysis, first strand cDNA synthesis was performed using MMLV reverse transcriptase (Promega, WI, USA) with oligo dT primer (Invitrogen). For miRNA expression analysis, cDNA conversion was done by using QuantiMir RT kit (System Biosciences, CA, USA). Quantitative real-time PCR was performed using 1X SYBR green PCR master mix in ABI 7500 Fast Real-time PCR system (Applied Biosystems, CA, USA). For mRNA expression analysis, the primers were 5’-TGCAAAGCTGCCAGTGAAGT-3’ (Forward) and 5’-GCCAAAGGACCACACATCTGA-3’ (Reverse) for c-MET [46] and 5’-CCCTGCCGCTTCCAGAATGCTT-3’ (Forward) and 5’-AAGGAGGGATCAGCCGCAGAAG-3’ (Reverse) for C9orf3. For miR-23b, the mature sequence of miR-23b was used as the forward primer and the 3’ universal reverse primer was provided by the QuantiMir RT kit. Real-time PCR analysis with primers specific for β-actin and human U6 RNA were also performed as internal control for mRNAs and miR-23b respectively. Relative standard curve (2^-ΔΔCt^) method was used to determine the relative mRNA and miRNA expressions of miR-23b.

### Western blot analysis

Western blot analysis was performed as previously described [27]. Primary antibodies against MET (L41G3), phosphor-MET (Tyr1234/1235), Akt (pan) (11E7), phosphor-Akt (Ser473), phosphor-Bad (Ser136) (Cell singling, MA, USA), Bad (C-20) (Santa Cruz Biotechnology, CA, USA) and β-actin (Sigma, MO, USA) were used.

### Ectopic miR-23b expression and inhibition

The upregulation or downregulation of miR-23b expression in cells was performed as previously described [27]. In each experiment, empty vector control or anti-miR negative control (Ambion) were also used in parallel.

### siRNA transfection

c-MET siRNA duplex with the sense sequence of 5’-AGAAUGUCAUUCUACAUGAGC-3’ [47], Drosha siRNA duplex with the sense sequence of 5’-AACGA GUAGGCUUCGUGACUU-3’ or p53 siRNA duplex with the sense sequence of 5’-GACUCCAGUGGUAA UCUAC-3’ [22] were transfected into cells with the aid of Lipofectamine 2000 (Invitrogen) for 24 h.

### Construction of c-MET 3’-UTR reporter vector

Segments of c-MET 3’-UTR containing the predicted miR-23b recognition sequence were amplified and subcloned into the SpeI and HindIII sites of the pMIR-REPORT miRNA expression reporter vector (Ambion). The primers were 5’-GCACTAGTACTGATGGTGTCATTCACCC-3’ (Forward) and 5’-AAAAGCTTTGTATTGCCTATAAATATTCTTC-3’ (Reverse). The construct was designated as pMIR-REPORT/cMET3’UTR.

### Luciferase reporter assay

The cells were seeded in a 24-well plate for 24 h and then transfected with the c-MET 3’-UTR reporter constructs together with miR-23b precursor vector or anti-miR-23b inhibitor for 24 h. At the same time, control vector containing the Renilla luciferase pRL-CMV was also included in the transfection for monitoring the transfection efficiency. The firefly luciferase activity was measured using the dual-luciferase reporter assay system according to the manufacturer's protocol (Promega). Relative luciferase activity was first normalized with Renilla luciferase activity and then compared with those of the respective control.

### Bisulfite modification and methylation-specific PCR (MSP) analysis of C9orf3 promoter region

Genomic DNA was extracted using DNAzol reagent and denatured by 0.2 M sodium hydroxide (NaOH) at 50°C for 10 min. Samples (1 μg) were incubated under mineral oil with freshly prepared 10 mM hydroquinone and 3 M sodium bisulfite (pH 5) at 50°C for 16 h. Modified DNA was then purified and desulfonated with 0.3 M NaOH, followed by ethanol precipitation and water elution. MSP was then carried out using methylation-specific primers (Forward: 5’-TTTGAGTAGTTGGGATTATAGGTGT-3’ and reverse: 5’-ACTTTAAAAAACCAAAACAAACAAA-3’) or unmethylation-specific primers (Forward: 5’-TTTCGAGTAGTTGGGATTATAGGC-3’ and reverse: 5’-CTTTAAAAAACCGAAACAAACGA-3’) for amplifying methylated and unmethylated CpG islands in the C9orf3 promoter region respectively. The PCR was started at 95°C for 5 min, then 15 touchdown cycles (30 s at 95°C, 45 s at 66.5°C with 0.5°C decrease each cycle and 60 s at 72°C), and 35 cycles with annealing temperature at 59°C, followed by a final extension at 72°C for 7 min. PCR products were then resolved in 2% agarose gel. The band intensity was analyzed by ImageJ software (version1.41o). The percentage of C9orf3 promoter methylation was calculated as M x 100% / (U+M). U: band intensity of the methylated PCR products; M: band intensity of methylated PCR products.

### Genomic bisulfite DNA sequencing

Bisulfite-modified genomic DNA was subjected to PCR for amplification using the primers specific for C9orf3 promoter region (Forward: 5’-GTTTAGGTTGGAGTGTAGTGG-3’ and reverse: 5’-AAAAACAAACATTAAATATAAAAAAAA-3’). The PCR was started at 94°C for 2 min, then 40 cycles of 94°C for 30 s, 54°C for 30 s and 72°C for 30 s, followed by a final extension at 72°C for 5 min. The amplification products were resolved in 1.2% agarose gel and then subcloned into the pGEM-T Easy vector (Promega). Five random positive clones were picked up for DNA sequencing.

### Annexin V binding assay

Cells were trypsinized and resuspended in Annexin V binding buffer (pH 7.4) containing 10 mM HEPES, 140 mM NaCl and 2.5 mM CaCl_2_ after washing. Cells were then stained with Annexin V-GFP and propidium iodide in the dark at room temperature for 15 min. Cell populations were analyzed by flow cytometry using BD FACSCanto flow cytometer (BD Biosciences) and data were analyzed by WinMDI 2.9.

### MTT assay

Cells were seeded in a 96-well plate and then subjected to transient transfection for 24 h. After 72 h of paclitaxel treatment, the cells were incubated with 50 μl of 1 mg ml^-1^ 3-(4,5-dimethylthiazol-2-yl)-2,5-diphenyltetrazolium bromide (MTT) (Sigma-Aldrich Co.) in PBS for 3 h. The formazan that formed was then solubilized by adding 150 μl of dimethyl sulfoxide. The absorbance was read at 570 nm using a FLUOstar Galaxy plate reader (BMG Labtech, Offenburg, Germany).

### Statistical analysis

SPSS software, version 17 (IBM Corp., Armonk, NY, USA), was used to perform statistical tests. Data are presented as the mean ± standard deviation. A two-tailed Student's t-test was used to test the differences in sample means for data with normally distributed means. A p-value of <0.05 was considered to be statistically significant.

## SUPPLEMENTARY MATERIALS FIGURES AND TABLES


