# Supplementary material for: Human papillomavirus type 16 E6 suppresses microRNA-23b expression in human cervical cancer cells through DNA methylation of the host gene C9orf3
Source: Oncotarget. 2017 Jan 6;8(7):12158–73. doi: 10.18632/oncotarget.14555 (PMC5355333; doi:10.18632/oncotarget.14555)
Supplement: Supplementary file 1 [file oncotarget-08-12158-s001.pdf]

Human papillomavirus type 16 E6 suppresses microRNA-23b expression in human cervical cancer cells through DNA methylation of the host gene C9orf3

SUPPLEMENTARY FIGURES

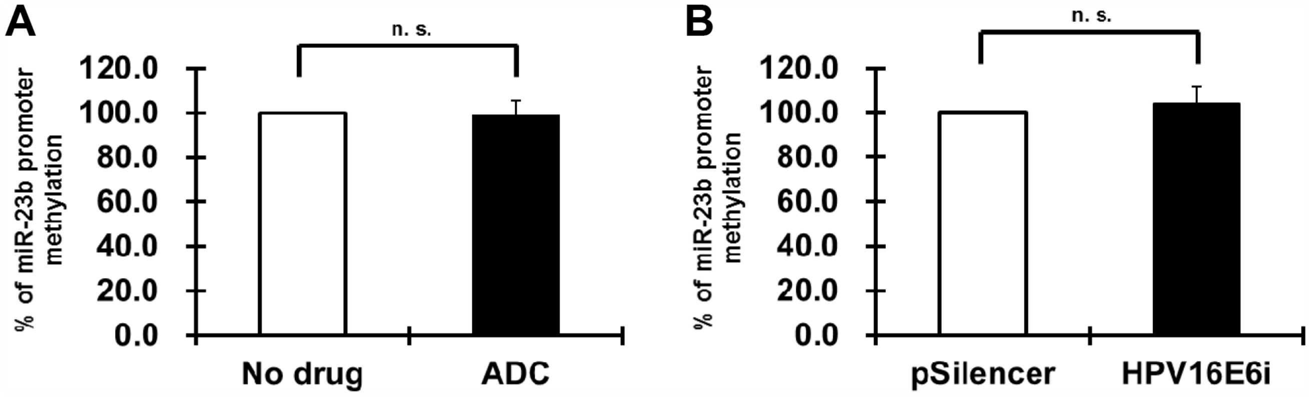

Supplementary Figure 1: The methylation status of the miR-23b promoter region in SiHa cells. A. the cells were either untreated (no drug) or treated with 5 µM 5-aza-2'-deoxycytidine (ADC) for 24 h. Bar chart showing the percentage of miR-23b promoter methylation using methylation specific PCR (MSP) analysis. Mean ± SD. n.s. = not significant. **B.** the cells were transiently transfected with pSilencer/HPV16E6siRNA (HPV16E6i) or pSilencer empty vector (pSilencer) for 24 h. Bar chart showing the percentage of miR-23b promoter methylation using methylation specific PCR (MSP) analysis. Mean ± SD. n.s. = not significant.

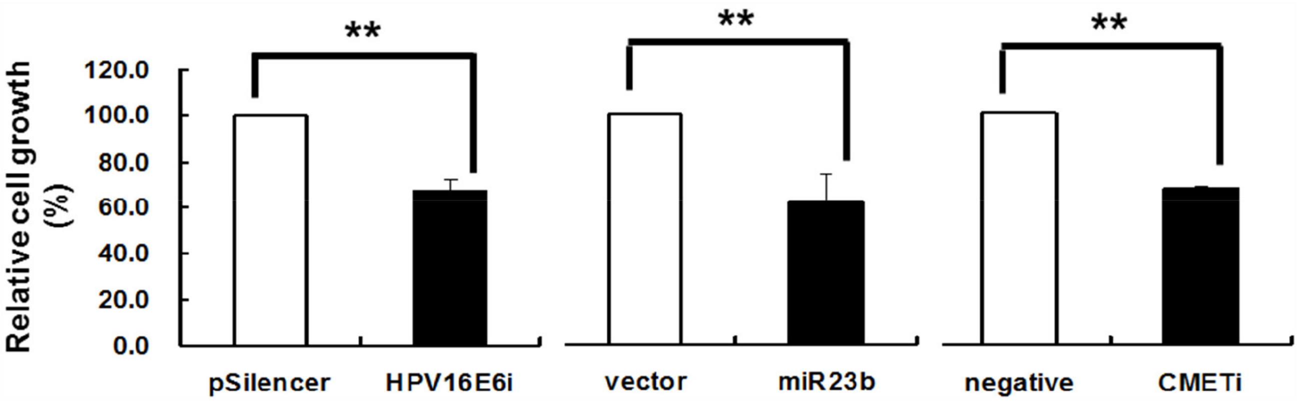

Supplementary Figure 2: The effect of HPV-16 E6, miR-23b and c-MET on cell growth of SiHa cells. The cells were transiently transected with pSilencer/HPV16E6siRNA (HPV16E6i), miR-23b precursor vector (miR23b) or c-MET siRNA (cMETi) for 24 hours. The cells were transfected with empty pSilencer vector (pSliencer), empty vector (vector) or negative siRNA (negative), respectively, in parallel. Cell growth was measured by MTT assay after 72-hour incubation. Results were average from at least three separate experiments. Mean ± SD. **p<0.01.

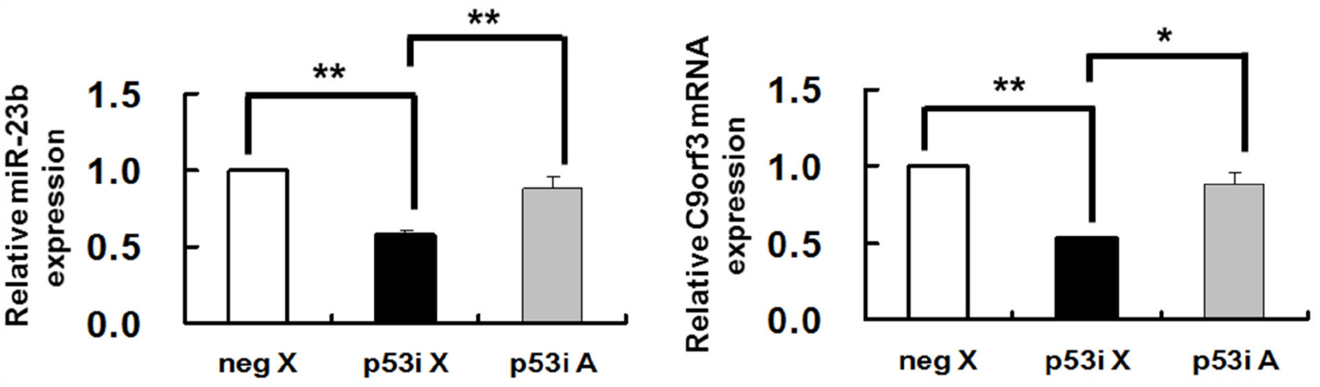

Supplementary Figure 3: The effect of p53 and 5-aza-2'-deoxycytidine on miR-23b and C9orf3 mRNA expressions in SiHa cells. The cells were transiently transfected with p53 siRNA (p53i) or negative siRNA (neg) for 24 h followed by 24-hour treatment of 5-aza-2'-deoxycytidine (A). X: no drug control. miR-23b and C9orf3 mRNA expression levels were then determined by quantitative RT-PCR analysis. Results were average from at least three separate experiments. Mean ± SD. *$p<0.05$, **$p<0.01$.
